# Quaternization drives spleen-to-lung tropism conversion for mRNA-loaded lipid-like nanoassemblies

**DOI:** 10.7150/thno.90071

**Published:** 2024-01-01

**Authors:** Yixuan Huang, Jiacai Wu, Sanpeng Li, Zhen Liu, Zhenghua Li, Boping Zhou, Bin Li

**Affiliations:** 1 Department of Infectious Disease, Shenzhen People's Hospital, The First Affiliated Hospital of Southern University of Science and Technology & The Second Clinical Medical College of Jinan University, Shenzhen 518020, China.; 2 School of Medicine, Southern University of Science and Technology, Shenzhen, 518055, China.

**Keywords:** quaternization, lipid-like nanoassembly, systemic mRNA delivery, lung targeting, ultra-high selectivity

## Abstract

**Background:** As the overwhelming majority of advanced mRNA delivery systems are preferentially accumulated in the liver, there is an accelerating growth in the demand for the development of non-liver mRNA delivery platforms.

**Methods:** In this study, we prepared cationic lipid-like nanoassemblies through a N-quaternizing strategy. Their physicochemical properties, in vitro mRNA delivery efficiency, and organ tropism in mice were investigated.

**Results:** Introduction of quaternary ammonium groups onto lipid-like nanoassemblies not only enhances their mRNA delivery performance in vitro, but also completely alters their tropism from the spleen to the lung after intravenous administration in mice. Quaternized lipid-like nanoassemblies exhibit ultra-high specificity to the lung and are predominantly taken up by pulmonary immune cells, leading to over 95% of exogenous mRNA translation in the lungs. Such mRNA delivery carriers are stable even after more than one-year storage at ambient temperature.

**Conclusions:** Quaternization provides an alternative method for design of new lung-targeted mRNA delivery systems without incorporation of targeting ligands, which should extend the therapeutic applicability of mRNA to lung diseases.

## Introduction

Lipid nanoparticles (LNPs) represent advanced nucleic acid delivery systems that have been used for clinically approved siRNA- and mRNA-based therapeutics 1-6. The vast majority of LNPs that are already being used in preclinical and clinical trials, however, are accumulated in the liver following intravenous administration, limiting their further therapeutic applications 4-13. The most prevalent explanation on the mechanisms of hepatic tropism is that LNPs adsorb apolipoprotein E during circulation in blood and boost internalization by hepatocytes via low-density lipoprotein receptor (LDLR)-mediated endocytosis 14-19. Nowadays, non-liver mRNA delivery strategies are increasingly being deployed to expand the application scope of LNPs for treating non-liver diseases. These strategies include rational design of new delivery vehicles 20-35, adjustment of the component ratios 36, incorporation of targeting ligands or additional charged components 37-42, and local delivery like inhalation, gastric, intracerebral, and intravesical injections 43-51.

To date, only a few delivery systems have been reported for targeted delivery of mRNA to the lung after systemic injection, mainly involving polymer nanoparticles 20-23, LNPs 28, 39-42, and their hybrids 24-27. mRNA delivered by polymer or lipid-polymer hybrids is more prevalent and often considered easier to reach non-liver tissues than that encapsulated in LNPs, probably because of the unique role of the liver in lipid absorption, transport, and metabolism 52. Currently, multiple strategies including functionalization of polymers, chemical structure modifications, and incorporation of positively charged components, have been adopted to achieve systemic mRNA delivery to the lung 20-28, 39-41. Despite the promise of such attempts, approaches that increase the organ selectivity or reduce the complexity of mRNA delivery systems deserve further exploration.

**Figure 1:**
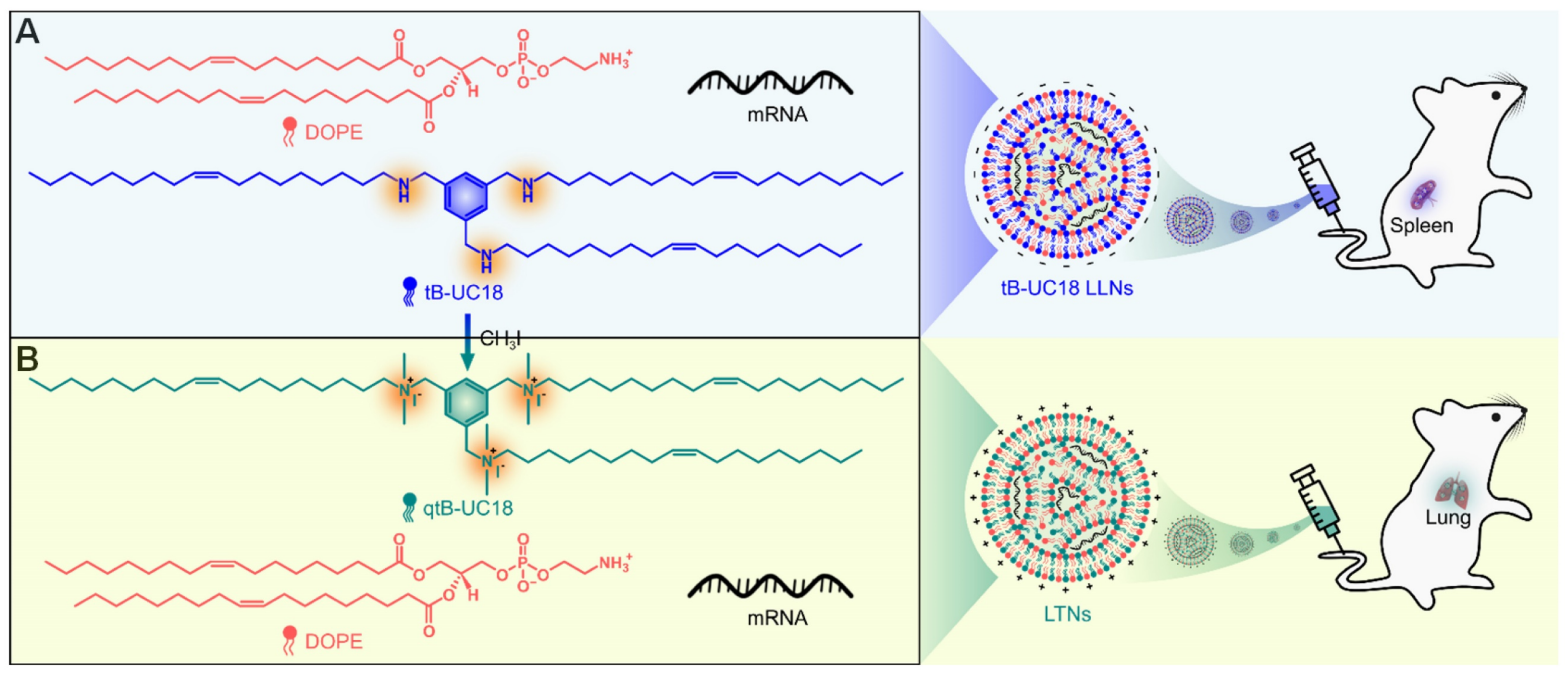
Schematic of tropism conversion of mRNA-loaded lipid-like nanoassemblies from the spleen to the lung via quaternization. The quaternized tB-UC18 derivative (qtB-UC18) in (B) was obtained through the N-alkylation reaction of secondary amines of tB-UC18 in (A) with iodomethane.

tB-UC18 is a novel lipid-like compound bearing three ionizable secondary amines adjacent to a core benzene ring 31. In our recent work, we have demonstrated that tB-UC18 combined with helper lipids 1,2-dioleoyl-sn-glycero-3-phosphoethanolamine (DOPE) was capable of self-assembling into lipid-like nanoassemblies (LLNs) for systemic mRNA delivery 31. Of all organs tested, mRNA loaded into tB-UC18 LLNs could be specifically delivered to the spleen after intravenous injection in mice (Figure 1A) 31. In this study, the secondary amines of tB-UC18 were quaternized (termed qtB-UC18) to obtain the cationic head groups (Figure 1B). qtB-UC18 formulated with DOPE enabled targeted mRNA delivery to the lung with ultra-high selectivity (Figure 1B), leading to over 95% of mRNA translation in the lung. Moreover, the newly developed mRNA delivery reagent remained active after more than one-year storage at room temperature. Overall, simple structural changes in the head groups of ionizable lipids provide a potential strategy for reprogramming the tissue tropisms of mRNA delivery systems.

## Materials and Methods

### Reagents

The helper lipid DOPE was ordered from Avanti Polar Lipids. The Laurdan reagent and chloropromazine (CPZ) were purchased from Macklin Inc. Filipin was purchased from Meilunbio. CH_3_I and 5-(N-ethyl-N-isopropyl)-amiloride (EIPA) were purchased from Aladdin. Lipofectamine 2000 (Lipo2K) was from Thermo Fisher Scientific. Triton X-100, fetal bovine serum, Eagle's Minimum Essential Medium, Dulbecco's Modified Eagle's Medium (high glucose), and RNase A were purchased from Sangon Biotech. One-Lumi Firefly Luciferase Reporter Gene Assay Kit, fluorescent probes DiD, ACK lysis buffer, and Ultrapure Water (BeyoPure, DNase/RNase-Free, Sterile) were from Beyotime Biotechnology. CD31-FITC and CD45-PE antibodies were ordered from BioLegend. Firefly luciferase (FLuc) mRNA and enhanced green fluorescent protein (eGFP) mRNA were from APExBIO. The purity of mRNA was determined by absorbance at 260 and 280 nm and by 1% agarose gel electrophoresis. DNase I and collagenase D were purchased from Solarbio. Cy5-labelled oligo(dT)_17_ was obtained from Genscript. Other chemical reagents in their analytic grades were used throughout without further purification.

### Synthesis of quaternized lipid-like compounds

The parent lipid-like compound, tB-UC18, was synthesized as described previously 31. To a round-bottom flask was added an ethanol solution (17 mL) of tB-UC18 (0.2 g, 0.2 mmol), CH_3_I (0.6 g, 4.2 mmol), and K_2_CO_3_ (0.12 g, 0.9 mmol). The reaction mixture was allowed to stir at 40 °C for 72 h, poured into a separating funnel containing H_2_O, and extracted three times with CH_2_Cl_2_. The combined organic layers were dried over MgSO_4_, filtered, and evaporated to afford qtB-UC18 as a white waxy oil. The purified qtB-UC18 was characterized by ESI-MS and ^1^H NMR.

### Preparation of quaternized lipid-like nanoassemblies

qtB-UC18 and DOPE dissolved in ethanol at the indicated molar ratios were diluted in nuclease-free water at a volume ratio of 1:9 (used for preparation of low-concentration formulations for in vitro studies) or 3:7 (for high-concentration formulations used for in vivo studies). The dispersed solution was then added to FLuc mRNA or eGFP mRNA solution at the indicated N:P ratios (N in qtB-UC18 and P in mRNA) under repeated pipetting. After rapid mixing, all formulations were kept at room temperature for 10 min prior to use. To compare the delivery efficiency, the final concentration of mRNA in different formulations was fixed as 0.02 mg mL^-1^ for in vitro studies and 0.05 mg mL^-1^ for in vivo experiments. To test the long-term stability, stock solutions containing equimolar qtB-UC18 and DOPE in an ethanol/water solvent were stored at ambient temperature for different time points and used to prepare LLNs. The in vitro delivery efficiency of the resulting LLNs was then evaluated by the methods described below.

### Characterizations of quaternized lipid-like nanoassemblies

The hydrodynamic size, polydispersity index, and zeta potential of formulations prepared at various N/P ratios were measured at 25 °C using dynamic light scattering (DLS, Brookhaven Instruments Corporation, 90Plus PALS). The generalized polarization for each formulation was quantified by Laurdan fluorescence as previously described 53, 54. The encapsulation efficiencies of some formulations were determined by the RiboGreen assay 55. The heat resistance of lung-targeted nanoparticles (LTNs) termed below was estimated by measurement of the hydrodynamic size using DLS under built-in temperature gradients with values of 25, 30, 35, 40, 45, 50, 55, and 60°C. The morphology of LTNs was assessed by transmission electron microscopy (Hitachi, HT7700). Turbidity analysis of LTNs in 10% FBS was determined by monitoring the change of absorbance at 660 nm as previously described 31, 56.

### Cell line and cell culture

Human embryonic kidney 293T cells were purchased from Stem Cell Bank, Chinese Academy of Sciences. Human cervical cancer HeLa cells, T lymphocyte Jurkat cells, acute monocytic leukemia THP-1 cells, and mouse embryonic fibroblast NIH/3T3 cells were purchased from Beyotime. All cells were maintained in ATCC-recommended medium supplemented with 10% (v/v) fetal bovine serum at 37 °C in a 5 % CO_2_ incubator.

### Assessment of mRNA delivery efficiency in vitro

293T cells were seeded onto 96-well plates at 20,000 cells per well. NIH/3T3 and HeLa cells were seeded at 10,000 cells per well. Jurkat and THP-1 cells were seeded at 40,000 cells per well. All cells were incubated overnight and then treated with formulations containing 200 ng of FLuc mRNA at 37 °C for 24 h. Cells were lysed and assayed for luciferase activity with One-Lum Firefly Luciferase Reporter Gene Assay Kit according to manufacturer's instructions. In the fluorescence imaging experiment, eGFP mRNA instead of FLuc mRNA was formulated into nanoassemblies using the above methods. The green fluorescent signal from cells treated with formulations for 24 or 48 h was detected by fluorescence inverted microscopes (Nikon ECLIPSE Ts2R or Zeiss Axio Vert A1).

### Gel electrophoresis

To confirm the interaction between nanocarriers and mRNA, formulations were loaded to 1% agarose gel, run in Tris-acetate-EDTA buffer at 120 V for 30 min, and imaged using an imaging system (Thermo Fisher Scientific, iBright FL1000). For the RNase degradation assay, LTNs were exposed to RNase A treatment (10 ng ml^-1^) for different time periods before gel electrophoresis.

### In vitro hemolytic effect

qtB-UC18 alone or combined with an equimolar amount of DOPE were incubated with 9 volumes of human red blood cells (4%, v/v) at various concentrations for 60 min at 37 °C. After centrifugation, the supernatant was quantified spectrophotometrically at a specific wavelength of 540 nm using a microplate reader (BioTek, Synergy LX). Triton X-100 (1%) and PBS were used as a positive and negative control, respectively.

### Cellular uptake and internalization

Flow cytometric analysis of cellular uptake mediated by two optimized formulations identified in vitro were carried out as described previously 57. In brief, Cy5-labelled mRNA was prepared by annealing an eightfold molar excess of Cy5-tagged oligo(dT)_17_ to the mRNA poly(A) tail and formulated into nanoassemblies as described above. 293T cells seeded at a 96-well plate were treated with or without various endocytosis inhibitors at the final concentrations of 10 μg mL^-1^ CPZ, 1 mg μL^-1^ filipin, and 10 μg mL^-1^ EIPA for 30 min at 37 °C prior to adding formulations. Cells were then exposed to nanoassemblies containing Cy5-labelled mRNA for 4 h at 37 °C or 4 °C, washed three times, and suspended in PBS for flow cytometric analysis (Agilent, NovoCyte).

### Animal experiments

Animal care and experimental protocols were approved by the Institutional Animal Care and Use Committee of Shenzhen People's Hospital in accordance with the guidelines for the care and use of laboratory animals. DiD-incorporated LTNs were generated with our previously reported method 31. FLuc mRNA-loaded formulations or DiD-incorporated LTNs were injected intravenously into female 8-week-old C57BL/6J mice at a mRNA dose of 0.5 mg kg^-1^ and sacrificed 4 h after injection. For bioluminescent imaging, mice were injected intraperitoneally with D-luciferin potassium salt (150 mg kg^-1^) 10 min prior to in vivo and ex vivo imaging (PerkinElmer, IVIS Spectrum) 55. Blood samples and major organs were collected for routine blood tests and histological staining, respectively. With regard to florescence imaging, florescence signals from excised organs were detected by an in vivo imaging system (VISQUE Invivo Smart-LF) with an excitation wavelength of 710 nm and an emission filter of 780 nm.

### Flow cytometry

Lung tissues from mice receiving DiD-incorporated LTNs were collected 4 h following injection and subjected to dissociation with collagenase I (final concentration 5 mg mL^-1^) and DNase I (final concentration 1 mg mL^-1^) at 37 °C for 30 min. Cell suspensions were filtered with 70 μm cell strainers, incubated with ACK lysis buffer to remove red blood cells, and incubated with CD3-FITC, CD11c-PE, CD19-FITC, and F4/80-PE antibodies for 30 min at 4 °C. Flow cytometric data were acquired on a NovoCyte flow cytometer (Agilent) and analyzed with the NovoExpress software (Agilent).

### Analysis of the nanoparticle protein corona

Nanoparticles at the mRNA concentration of 0.05 mg mL^-1^ were incubated with an equal volume of mouse plasma at 37 °C for 30 min with gentle orbital shaking. Nanoparticle-protein corona complexes were then separated by ultracentrifugation according to the previously reported methods 28. Of these, one part was mixed with loading buffer and loaded onto a 10% precast protein gel for SDS-PAGE. Gels were stained with Coomassie Stain for 1 h and de-stained overnight prior to imaging with an imaging system (Thermo Fisher Scientific, iBright FL1000). The other part was analyzed by electrospray liquid chromatography-tandem mass spectrometry to quantify the protein corona composition as described previously 28.

### Statistical analysis

All data are presented as mean ± SEM. Unless specified otherwise, significant differences between two groups were evaluated by unpaired two-tailed Student's t-test. * indicates significant at a 0.05 probability level; NS indicates not significant at a 0.05 probability level.

## Results and Discussion

### Optimization of quaternized lipid-like nanoassemblies designed for mRNA delivery

The parent lipid-like compound tB-UC18 was obtained by a previously reported method 31. Its quaternized derivative, qtB-UC18, was obtained by introduction of methyl groups onto the nitrogen atoms of tB-UC18 via the *N*-alkylation reaction with iodomethane. The representative synthetic route to qtB-UC18 is shown in Figure 1. This reaction mixture was purified on column chromatography to afford qtB-UC18, which was characterized by HRMS (ESI) *m*/*z*: [M]^3+^ calcd for C_69_H_132_N_3_, 334.3468; found, 334.3612 (Figure S1) and ^1^H NMR (400 MHz, CDCl_3_, δ): 8.24 (s, 3H), 5.34 (t, *J* = 5.8 Hz, 6H), 4.96 (s, 6H), 3.35 (s, 18H), 2.01 (dd, *J* = 12.7, 6.7 Hz, 12H), 1.82 (s, 6H), 1.26 (d, *J* = 5.4 Hz, 72H), 0.88 (t, *J* = 6.7 Hz, 9H) (Figure S2).

**Figure 2:**
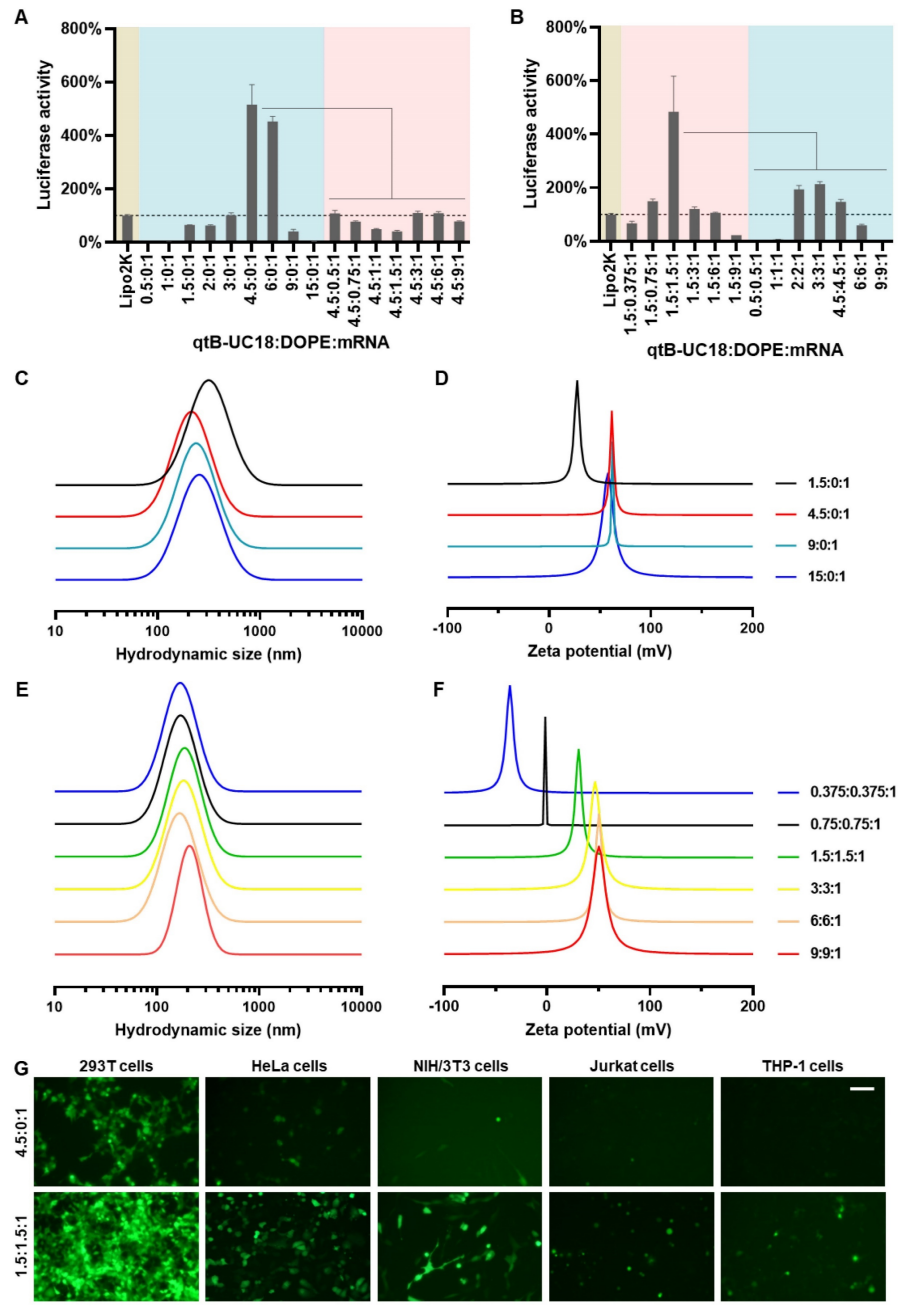
Optimization of qtB-UC18 LLNs for intracellular mRNA delivery. (A) Sequential optimization of formulation parameters in the order of N:P ratios, followed by molar ratios. (B) Sequential optimization of formulation parameters in the order of molar ratios, followed by N:P ratios. The formulation in (A) and (B) was expressed as qtB-UC18:DOPE:mRNA, where qtB-UC18:DOPE represented the molar ratio between qtB-UC18 and DOPE, and qtB-UC18:mRNA represented the N:P ratio between qtB-UC18 and mRNA. Luciferase activity of FLuc mRNA-loaded LLNs was normalized to the Lipo2k group. The light blue zone indicated N:P ratio optimization while the pink zone indicated molar ratio optimization. (C, D) The hydrodynamic size (C) and zeta potential (D) of qtB-UC18 LLNs formed through complexation between qtB-UC18 and mRNA. (E, F) The hydrodynamic size (E) and zeta potential (F) of qtB-UC18 LLNs formed through complexation between qtB-UC18/DOPE and mRNA. (G) Fluorescent images of cells incubated with eGFP mRNA-loaded qtB-UC18 LLNs for 48 h. Scale bar, 100 μm.

Initially, we attempted to load FLuc mRNA to binary complexes composed of qtB-UC18 and DOPE using a previously described ethanol dilution method 31. However, we found that such preparation failed to produce luminescence signals. We thus modified the procedure and observed that dispersion of qtB-UC18 into water prior to mixing with mRNA seem to be essential for effective mRNA delivery. In order to maximize the delivery efficiency, two optimization strategies were leveraged to prepare qtB-UC18 LLNs. For each strategy, two rounds of optimization were implemented with a fixed amount of mRNA. The first one was to optimize the N:P ratios before the addition of DOPE. qtB-UC18 displayed the highest activity when formulated with mRNA at the N:P ratio of 4.5:1 (Figure 2A). Of note, the activity was 5-fold higher than the commercial Lipofectamine 2000 (Figure 2A). After identifying the optimal N:P ratio, we investigated the influence of DOPE on qtB-UC18-mediated mRNA delivery. Formulations with a fixed N:P ratio (4.5:1) but varied molar ratios (qtB-UC18:DOPE) ranging from 4.5:0.5 to 4.5:9 were prepared and their activity was evaluated using the luciferase activity assay. Unexpectedly, qtB-UC18 formulated with DOPE dramatically decreased delivery efficiency compared with qtB-UC18 alone (Figure 2A).

In a separate experiment for the other strategy, we fixed the N:P ratio at 1.5:1, an optimal ratio previously used for tB-UC18, and investigated the effect of qtB-UC18 to DOPE molar ratios on mRNA delivery efficiency. The results in Figure 2B indicated that qtB-UC18 formulated with equimolar DOPE provided the superior performance for mRNA delivery, which was 5-fold higher than Lipofectamine 2000 (Figure 2B). To further explore N:P effects, formulations with an equimolar mixture of qtB-UC18 and DOPE were prepared at different N:P ratios. As indicated in Figure 2B, qtB-UC18 displayed the best performance just at N:P ratio of 1.5:1, when DOPE was present in the binary complexes. The lower and higher N:P ratios compromised delivery efficiency (Figure 2B). In comparison to qtB-UC18 alone, the presence of DOPE diminished the amount of qtB-UC18 used (Figure 2B). Taken together, superior mRNA delivery activity was achieved with qtB-UC18 alone (N:P ratio, qtB-UC18:mRNA = 4.5:1) or formulated with DOPE (molar ratio, qtB-UC18:DOPE = 1:1; N:P ratio, qtB-UC18:mRNA = 1.5:1). The encapsulation efficiencies determined by the RiboGreen assay were 57% and 97%, respectively.

For all one-component or two-component nanocarriers tested, they self-assembled into nanoparticles in the presence of mRNA with average hydrodynamic diameters of 150 nm ~ 300 nm (Figure 2C and 2E). With regard to the second parameter examined, we noticed that zeta potentials were positive when the N:P ratios for qtB-UC18:mRNA were greater than 1.5:1 regardless of one-component or two-component vehicles (Figure 2D and 2F). A decrease in N:P ratios for two-component nanocarriers led to the reversal of the surface charge from positive to negative (Figure 2F) and a reduction in the interaction between nanocarriers and mRNA (Figure S3). Nevertheless, a measurement of the generalized degree of polarization using Laurdan had shown that the N:P ratio did not correlate with the surface polarity (Figure S4). These results indicated that N:P ratios tested in this study had a profound influence on formulations' charges and complexation but not polarities.

In addition to 293T cells, we next tested the delivery performance of optimized formulations in other cell lines including HeLa, NIH/3T3, Jurkat, and THP-1 cells. Both formulations also enabled intracellular delivery of FLuc mRNA to HeLa and NIH/3T3 cells. The activity was moderate when assayed with hard-to-transfect Jurkat and THP-1 cells (Figure S5). Similar trend was observed for formulations loaded with eGFP mRNA (Figure 2G).

### In vitro evaluation of the optimized lipid-like nanoassemblies

**Figure 3:**
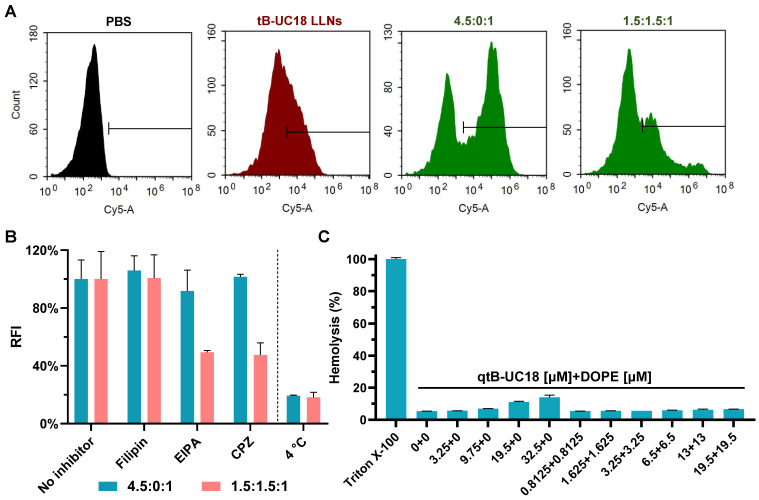
Cellular uptake and hemolytic activity of qtB-UC18 LLNs. (A) Representative flow cytometric histograms for analysis of cellular uptake mediated by different formulations. (B) The effects of endocytosis inhibitors and temperature on the cellular uptake of qtB-UC18 LLNs containing Cy5-labelled mRNA. The relative fluorescence intensity (RFI) of each group was normalized to the PBS (no inhibitor) group. (C) Hemolysis detection for single-component or dual-component carriers. The hemolytic activity was normalized to that of Triton X-100.

The optimal formulation compositions (qtB-UC18:mRNA = 4.5:1 and qtB-UC18:DOPE:mRNA = 1.5:1.5:1) identified above from two different optimization strategies were selected for more detailed in vitro studies. Flow cytometric analysis revealed that uptake percentages of Cy5-labelled mRNA by 293T cells were 31%, 56%, and 27%, respectively, when formulated into tB-UC18 LLNs and two optimized formulations (Figure 3A). Taking into account their delivery efficiencies, we speculated that the helper lipids DOPE in formulations may boost the release of mRNA from endosomal entrapment. An investigation into the mechanism of cellular uptake with various endocytosis inhibitors including filipin, EIPA, and CPZ revealed that all these inhibitors examined had little or no influence on mRNA uptake mediated by qtB-UC18 alone (Figure 3B). However, the internalization of mRNA encapsulated in binary complexes was significantly inhibited by EIPA or CPZ (Figure 3B). These observations reflected differences in uptake mechanisms between one- and dual-component qtB-UC18 nanocarriers and suggested that clathrin-mediated endocytosis and macropinocytosis were the predominant modes of entry into cells for nanocarriers containing equimolar qtB-UC18 and DOPE. Further low-temperature incubation confirmed that both formulations were internalized via an energy-dependent endocytic pathway (Figure 3B). Regarding in vitro hemolytic effect, qtB-UC18 alone or combined with DOPE only induced slight hemolysis when compared with PBS-treated red blood cells at pH 7.4, even at the maximum concentration tested, suggesting that the newly developed nanocarriers had great biocompatibility (Figure 3C).

### In vivo evaluation of efficacy, specificity, and safety of lipid-like nanoassemblies

**Figure 4:**
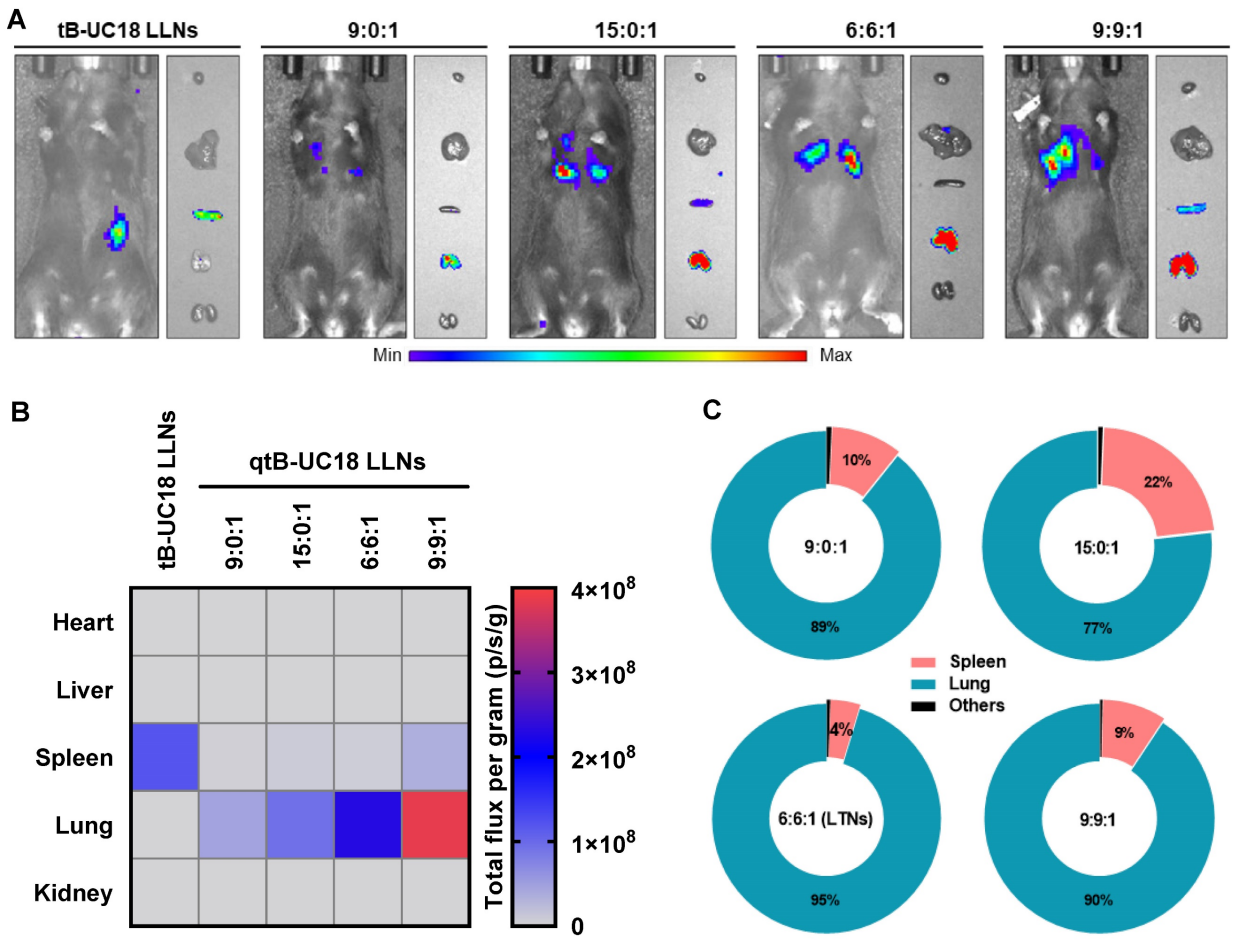
Delivery efficiency and specificity of formulations tested in the in vivo studies. (A) In vivo bioluminescence imaging of luciferase expression mediated by qtB-UC18 LLNs 4 h after intravenous injection. tB-UC18 LLNs served as a control. (B) Quantification of luciferase expression in each organ examined. (C) The percentage of luciferase expression in major organs.

We proceeded to animal experiments with the optimized formulations identified above (qtB-UC18:mRNA = 4.5:1 and qtB-UC18:DOPE:mRNA = 1.5:1.5:1). However, we found that mice receiving an intravenous injection of qtB-UC18 LLNs-encapsulated FLuc mRNA at 0.5 mg kg^-1^ did not give any detectable bioluminescence signals, implying that formulations developed from in vitro studies did not guarantee their in vivo potency 58, 59. Such result impelled us to adjust the N:P ratio by using a fixed amount of mRNA (0.5 mg kg^-1^) with varying molar ratios of nanocarriers containing qtB-UC18 alone or both qtB-UC18 and DOPE. In this case, we noticed that remarkable bioluminescence signals appeared in the lungs of mice at higher N:P ratios (Figure 4A). The maximum bioluminescence intensity was observed in the lungs for two types of formulations at the highest N:P ratio tested (Figure 4B). The rank order of specificity to the lung indicated that an increase in the N:P ratio of formulations compromised their lung-specific protein expression (Figure 4C). For qtB-UC18 LLNs with a qtB-UC18:DOPE molar ratio of 1:1 and a N:P ratio of 6:1 (hereinafter referred to as lung-targeted nanoparticles, LTNs), over 95% of luminescence signals originated from the lung (Figure 4C). The encapsulation efficiency of this formulation reached 99%. For the control group tB-UC18 LLNs, however, spleens were found to be almost the only target organ for mRNA translation (Figure 4A and 4B). These findings demonstrated that quaternization of the ionizable amine group in lipid-based formulations dramatically altered their tropism in mice.

**Figure 5:**
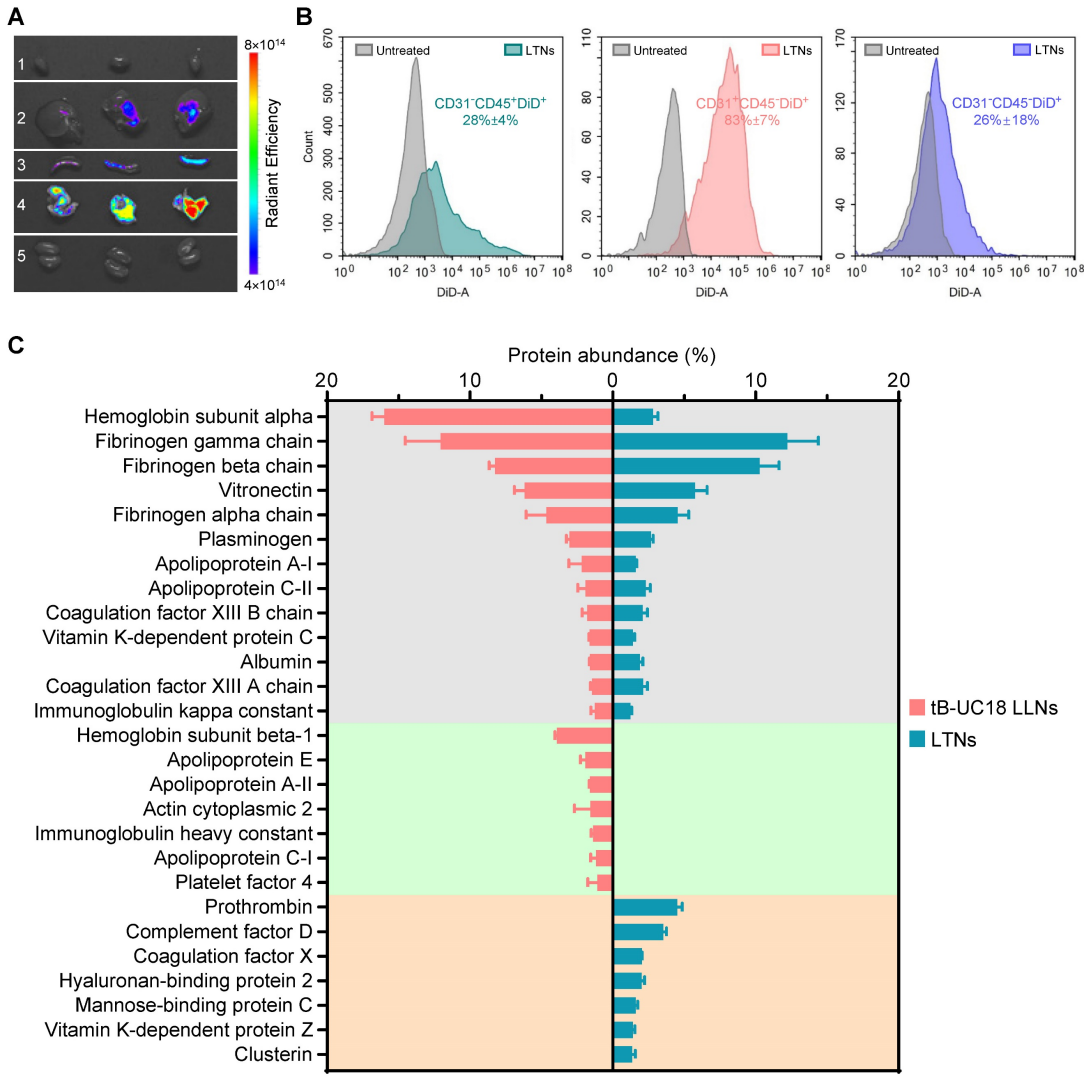
The bio-distribution of fluorescently labeled LTNs and the protein corona adsorption onto LTNs. (A) The bio-distribution of DiD-incorporated LTNs in the heart (line 1), liver (line 2), spleen (line 3), lung (line 4), and kidney (line 5) 4 h after intravenous administration. (B) Flow cytometric analysis of the percentage of DiD positive cells for each cell populations in the lung tissues from mice receiving DiD-incorporated LTNs. (C) Identification of the top 20 most abundant proteins adsorbed to formulations by LC-MS/MS.

To elucidate the specificity of LTNs, we conducted ex vivo fluorescence imaging with far-red fluorescent probes DiD-incorporated formulations. A strong fluorescence signal was found in the lungs over the other organs imaged (Figure 5A), which had substantial overlap with the bioluminescence signal obtained through ex vivo bioluminescence imaging (Figure 4A). These observations suggested the relevance of LTNs-mediated specific bio-distribution to mRNA translation. To further verify which kind of cells internalized nanoparticles, mouse lung samples were processed into single-cell suspensions for flow cytometric analysis. Subsequently, two gating strategies were exploited to identify DiD positive pulmonary immune cells, endothelial cells and other cells (Figure S6A). Among these DiD positive cell population, the majority (43%) were immune cells (CD31^-^CD45^+^) (Figure S6B). A further survey indicated that 28% of immune cells were DiD positive compared to 83% of endothelial cells (CD31^+^CD45^-^) (Figure 5B). As about half of the detected mouse pulmonary cells are immune cells (54%, Figure S6C), it was concluded that LTNs was predominantly delivered to immune cells in the lung.

Protein corona composition is considered as one of the key factors that affect the biodistribution of nanoparticles upon systemic administration 60. For conventional four-component 306-N16B LNPs developed for systemic lung-targeting mRNA delivery, the top three proteins in their corona are serum albumin, fibrinogen beta chain, and fibrinogen gamma chain 28. However, these proteins are replaced by vitronectin, serum paraoxonase, and apolipoprotein, respectively, in the presence of five-component lung-targeting SORT LNPs 18. In this study, we examined the adsorption of proteins onto the surface of our dual-component lipid-like nanoassemblies by SDS-PAGE and LC-MS/MS. LTNs displayed a different nanoparticle protein corona profile compared to that of tB-UC18 LLNs after polyacrylamide gel electrophoresis (Figure S7). Among the top 20 most abundant proteins identified by LC-MS/MS, hemoglobin subunit alpha was found to be the top protein binding to spleen-targeting tB-UC18 LLNs. Its abundance was 5.7-fold higher than that bound to LTNs (the gray zone, Figure 5C). Furthermore, tB-UC18 LLNs and LTNs had different abilities to selectively absorb their own proteins (the green zone vs the orange zone, Figure 5C). These findings suggested that quaternization was capable of driving alteration in protein corona composition and might account for their differences in organ tropism. It was worth mentioning that although fibrinogen gamma chain, fibrinogen beta chain, and vitronectin that were highly enriched in 306-N16B or SORT LNPs were identified as the three most abundant proteins in the corona of LTNs (the gray zone, Figure 5C), they should not be considered as the key factor for driving lung-specific mRNA delivery in view of their superior adsorption capacities for spleen-targeting tB-UC18 LLNs. Given that LTNs only consist of a binary mixture and do not contain the PEGylated lipids, in comparison to four- or five-component lung-targeting LNPs, we reasoned that the chemical composition of mRNA delivery carriers influences their protein corona profiles and targeted mRNA delivery.

We subsequently evaluated the safety of the LTNs by analyzing routine blood parameters from treated mice. No obvious impact was observed on hematology parameters except for slight elevation in monocyte subsets (Figure S8). Further hematoxylin and eosin staining revealed no apparent histopathological changes in the tissues examined (Figure S9). Aside from histopathological and hematological examination, we also investigated the effect of LTNs on the serum complement system C5a. Similarly, at two doses tested, no abnormalities occurred in complement activation fragment C5a compared with the PBS- or mRNA-treated control group (Figure S10).

### Assessment of stability of lung-targeted nanocarriers and nanoformulations

**Figure 6:**
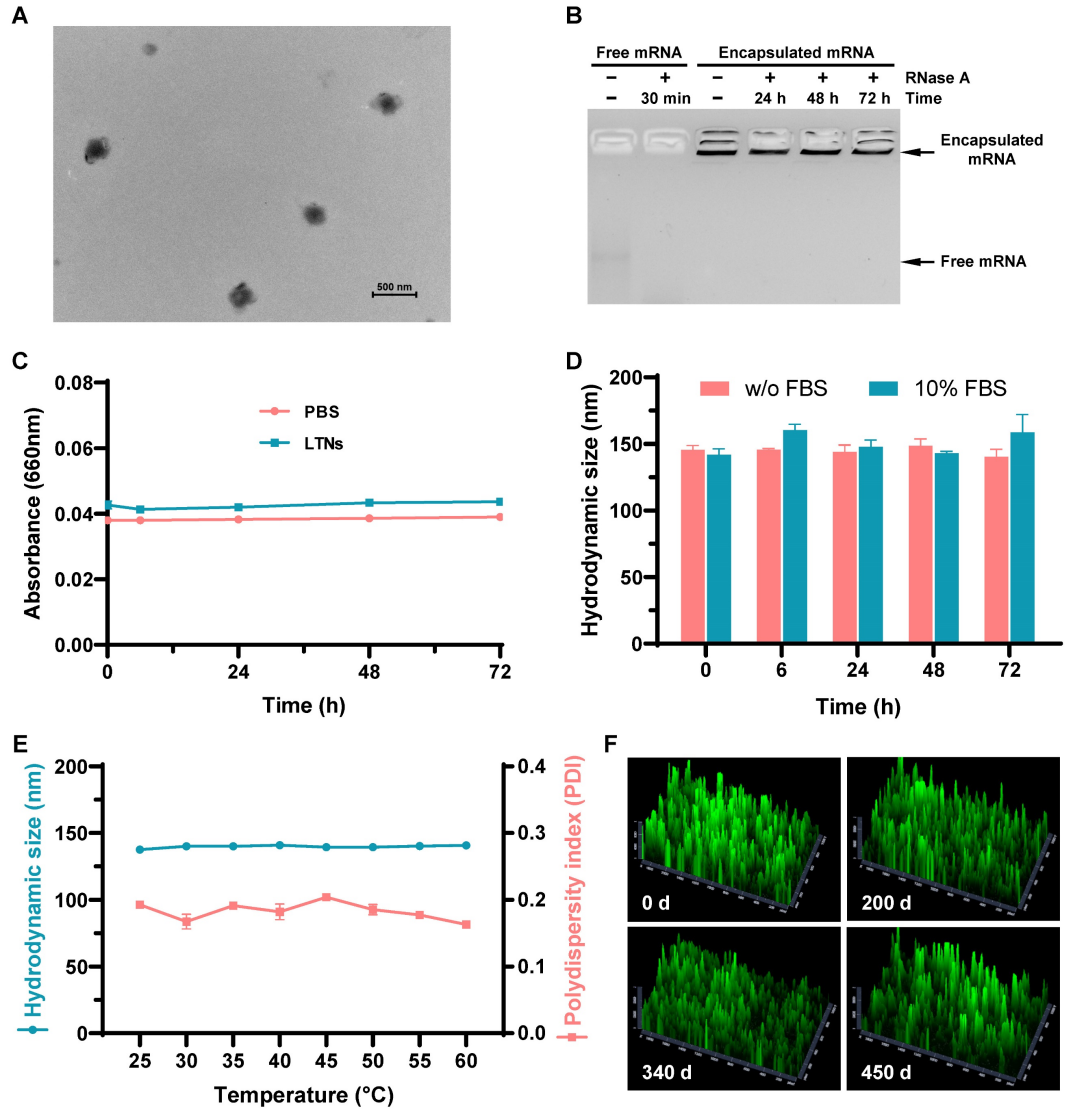
Stability of lung-targeted nanocarriers and nanoformulations. (A) The representative transmission electron micrograph showing the morphology of LTNs. (B) Assessment of the resistance of LTNs to RNase A-induced degradation through gel electrophoresis. LTN-encapsulated or free mRNA were incubated with RNase A at the indicated time points before electrophoresis. (C, D) Serum stability of LTNs determined by monitoring changes in turbidity (C) and hydrodynamic size (D). (E) The effect of temperature on the hydrodynamic size and polydispersity index of LTNs. (F) Assessment of the long-term stability of lung-targeted nanocarriers containing equimolar qtB-UC18 and DOPE after 200, 340, and 450 days of storage in an ethanol/water solvent at ambient temperature. The delivery performance was evaluated by encapsulating eGFP mRNA into stored nanocarriers and monitoring the green fluorescent signals originating from 293T cells after 24 h of treatment.

After establishing the optimal formulation parameters, we inspected the morphology of LTNs using transmission electron microscopy. qtB-UC18 mixed with DOPE formed nanoassemblies in the presence of mRNA with an average diameter of 200 nm (Figure 6A). As the stability of nanoparticles was a critical parameter for mRNA delivery system, we performed stability studies by incubating formulations with RNase A. Gel electrophoresis indicated that the naked mRNA was degraded within 30 min in the presence of RNase A, while mRNA encapsulated in nanoassemblies was stable over a period of 72 h (Figure 6B). Meanwhile, we estimated the serum stability by monitoring the changes of serum turbidity and found no serum-induced aggregation within 72 h (Figure 6C). This result was consistent with the unaltered hydrodynamic size of LTNs determined by dynamic light scattering (Figure 6D). Altogether, these data confirmed that formulations remained stable in the presence of RNase A or serum at room temperature. Furthermore, we tested the thermal sensitivity of LTNs and found that elevations in temperature did not cause remarkable fluctuation in hydrodynamic size (Figure 6E), reflecting the superior resistance of LTNs to high temperature-induced degradation.

Generally speaking, mRNA delivery carriers should be kept at refrigerator temperatures or even lower for long-term storage. For instances, almost all commercial mRNA delivery reagents should always be stored at 4 °C to maintain their high-efficiency transfection activity. To investigate the influence of storage time and temperature on the long-term chemical stability of lung-targeted nanocarriers, we evaluated their delivery efficiencies following storage at ambient temperature for 200, 340, and 450 days. During storage in ethanol/water solutions at room temperature, nanocarriers containing equimolar qtB-UC18 and DOPE showed no evident loss of delivery efficiency at the N:P ratio of 1.5:1, even after storage for 450 days (Figure 6F and Figure S11). This result revealed that the complexes were stable under ambient conditions with a shelf life of more than one year.

## Conclusion

In summary, a cationic lipid designed for selective delivery of mRNA to the lung was prepared via quaternization of a lipid-like compound previously used for splenic mRNA delivery. Sequential optimization of formulation parameters including the molar ratios between cationic lipids and helper lipids, and the N:P ratios between cationic lipids and mRNA molecules, enabled rapid identification of top-performing formulations. When applying to systemic mRNA delivery, however, two optimal formulations identified in vitro lost their activity in mice. Building on these results, we shifted our focus to higher N:P ratios tested in vitro. Formulations prepared at such ratios resulted in distinct protein expression profiles in organs, with one (termed lung-targeted nanoparticles, LTNs) exhibiting the highest specificity to the lung. In vivo living animal imaging revealed that more than 95% of LTN-formulated mRNA was specifically translated into proteins in the lung. The lung-targeted mRNA delivery reagent not only possessed high temperature resistance but also retained their activity after long-term ambient storage.

tB-UC18 LLNs are negatively charged nanoformulations which we previously used for spleen-selective mRNA delivery 31, while in this study, quaternization of the ionizable component altered formulations' protein corona composition and tissue tropism from the spleen to the lung after intravenous administration in mice. Such alteration might be attributed to the total reversal of surface charges of nanoassemblies. Collectively, minor tuning of ionizable amine groups enables the drastic improvement of lipid-based nanoparticle-mediated pulmonary mRNA expression, without incorporation of targeting ligands. The newly developed thermo-insensitive two-component nanocarrier reported here provides a highly selective pulmonary delivery platform for mRNA-based therapeutics with potential use in treating pulmonary diseases.

## Supplementary Material

Supplementary figures.

## Abbreviations

CPZ: chloropromazine
DOPE: 1,2-dioleoyl-sn-glycero-3-phosphoethanolamine
eGFP: enhanced green fluorescent protein
EIPA: 5-(N-ethyl-N-isopropyl)-amiloride
FLuc: firefly luciferase
LDLR: low-density lipoprotein receptor
LLN: lipid-like nanoassembly
LNP: lipid nanoparticle
LTN: lung-targeted nanoparticle
mRNA: messenger RNA
