# Supplementary material for: Quaternization drives spleen-to-lung tropism conversion for mRNA-loaded lipid-like nanoassemblies
Source: Theranostics. 2024 Jan 1;14(2):830–42. doi: 10.7150/thno.90071 (PMC10758058; doi:10.7150/thno.90071)
Supplement: Supplementary file 1 — Supplementary figures. [file thnov14p0830s1.pdf]

Supplementary materials

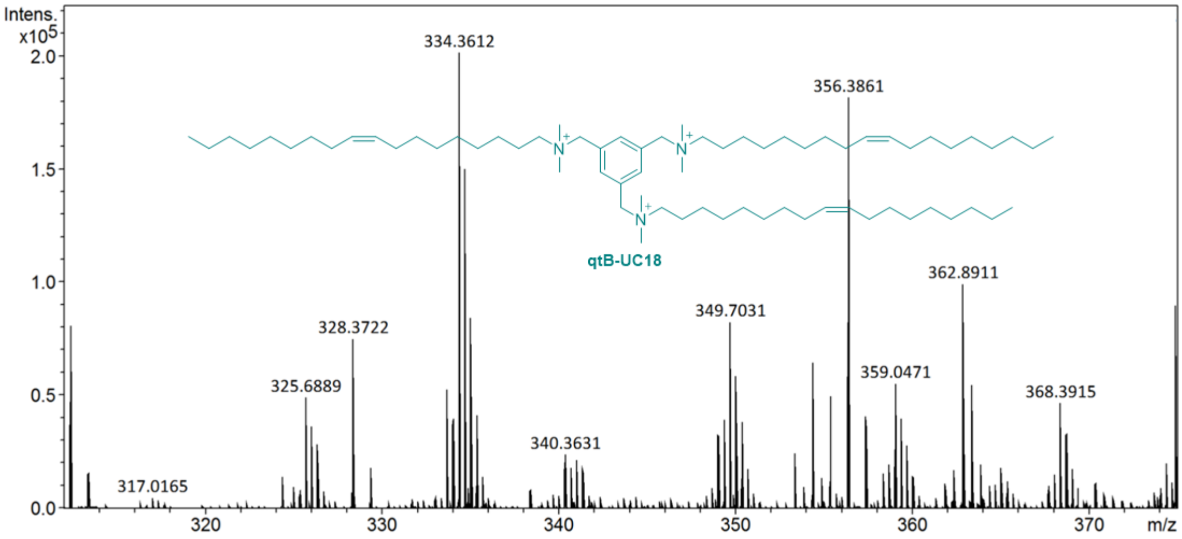

Figure S1. Positive ion ESI-MS spectrum of qtB-UC18.

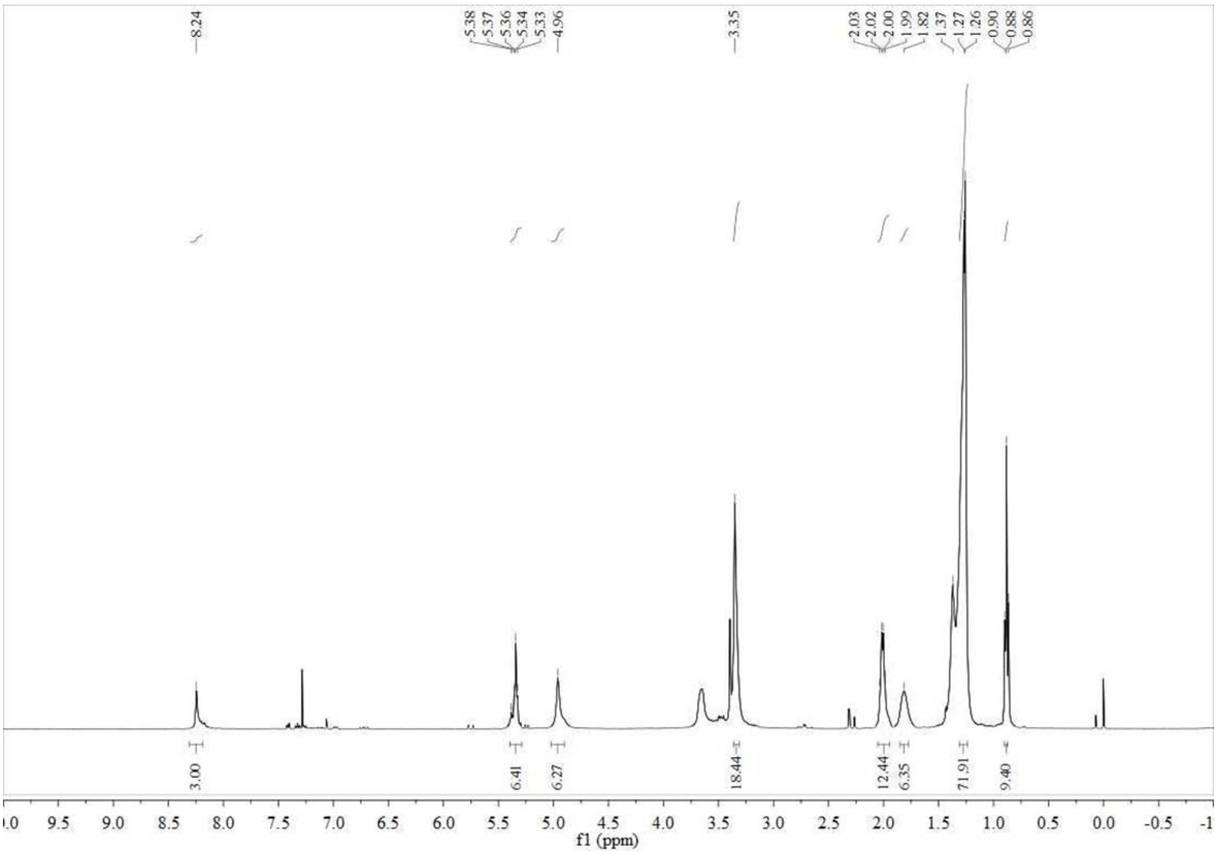

Figure S2. ^{1}H NMR spectrum of qtB-UC18.

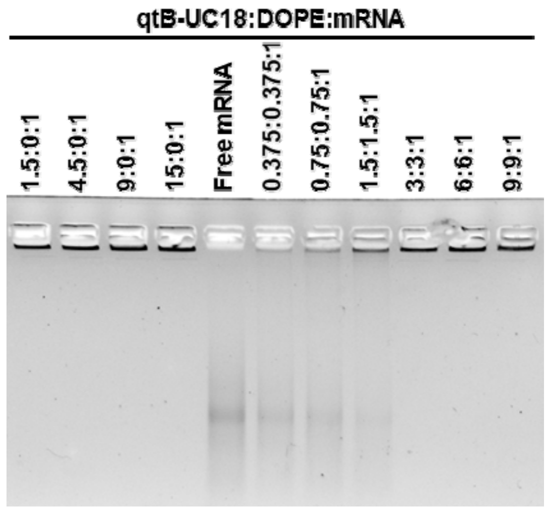

Figure S3. Confirmation of the interaction between nanocarriers and mRNA by gel electrophoresis.

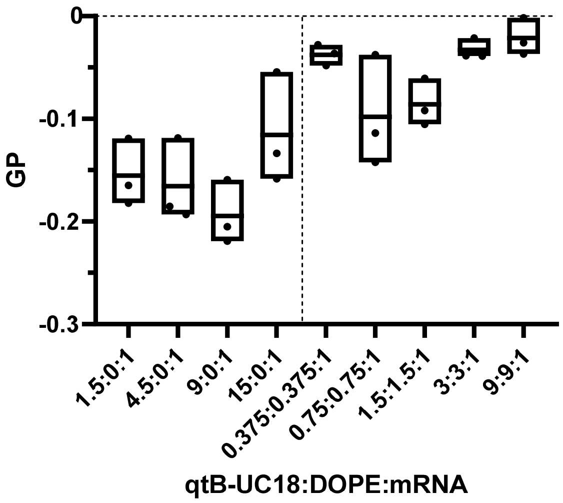

Figure S4. Measurement of the polarity of formulations using the laurdan generalized polarization method.

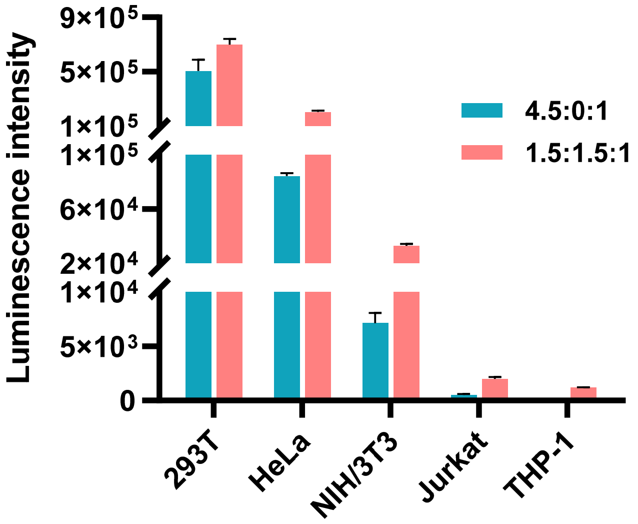

Figure S5. Intracellular delivery of FLuc mRNA mediated by two optimized formulations in 293T, HeLa, NIH/3T3, Jurkat, and THP-1 cells.

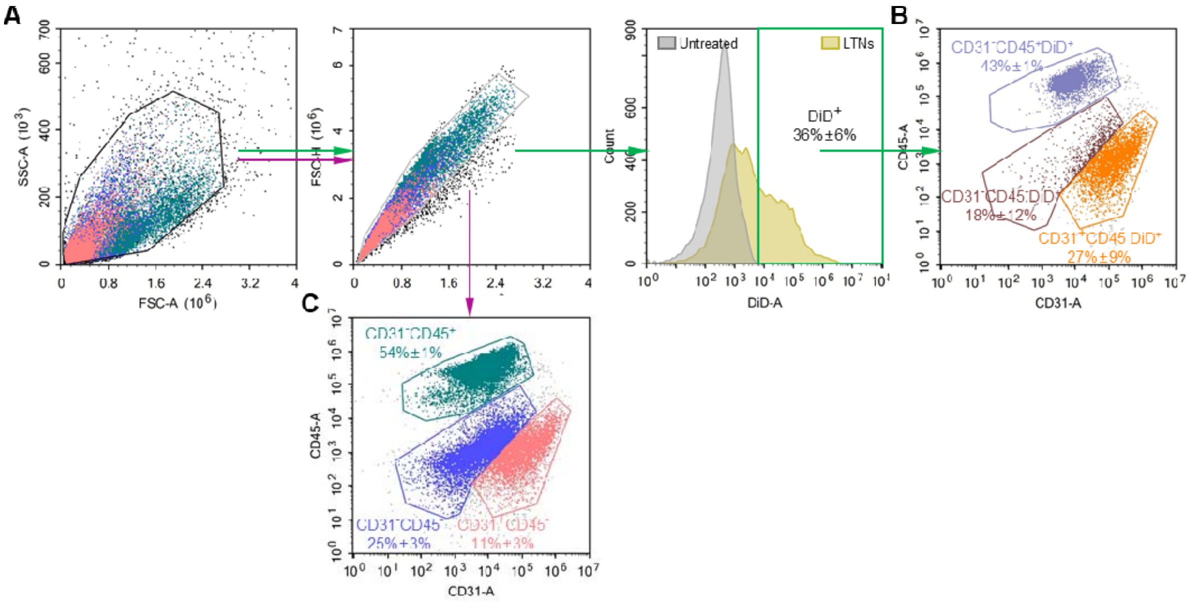

Figure S6. Flow cytometric analysis of DiD-incorporated LTNs in lung cells. (A) Two representative flow cytometric gating strategies for analysis of DiD positive pulmonary immune cells, endothelial cells, and other cells. (B) The percentage of DiD positive pulmonary immune cells, endothelial cells, and other cells in total DiD positive cell populations. (C) The percentage of each cell populations within lung cells.

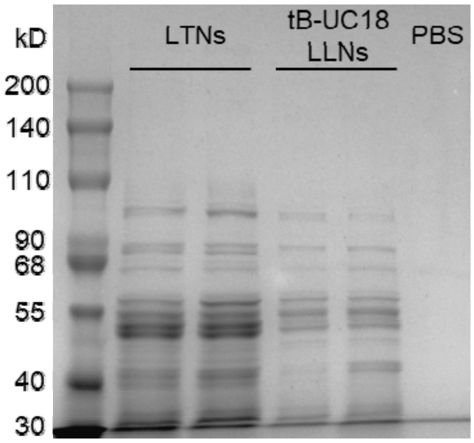

Figure S7. SDS-PAGE analysis of the protein corona bound to LTNs or tB-UC18 LLNs.

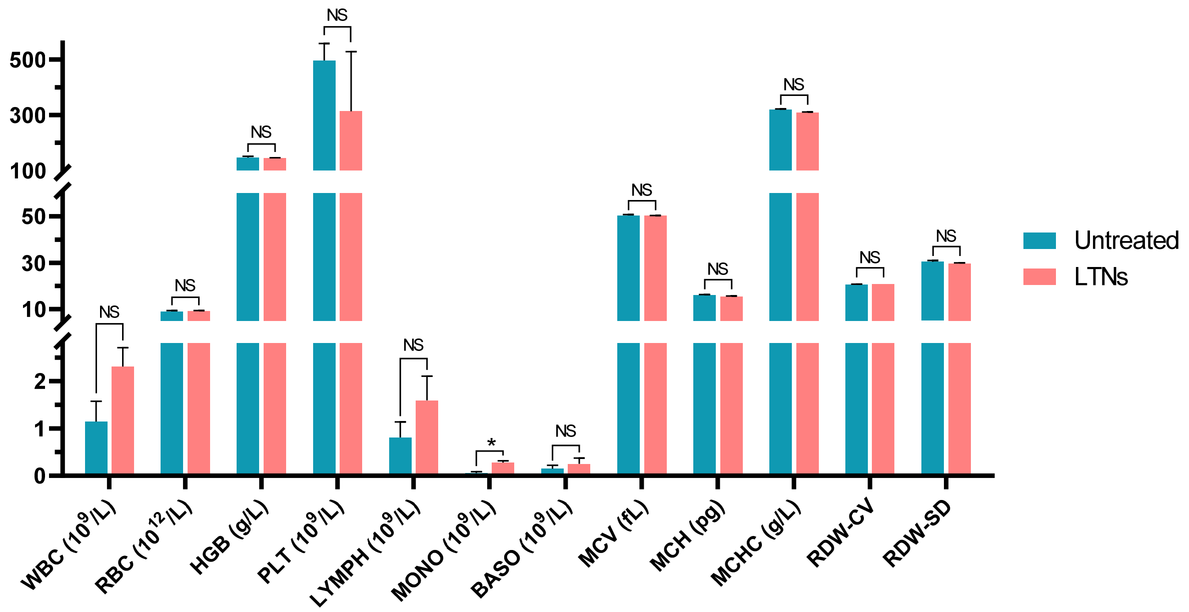

Figure S8. The effect of LTNs on routine blood parameters of mice receiving LTNs at a mRNA dose of 0.5 mg kg^{-1}. WBC, white blood cells. RBC, red blood cells. HGB, hemoglobin. PLT, platelets. LYMPH, Lymphocytes. MONO, Monocytes. BASO, Basophils. MCV, mean corpuscular volume. MCH, mean corpuscular hemoglobin. MCHC, mean corpuscular hemoglobin concentration. RDW-CV, red cell distribution width-coefficient of variation. RDW-SD, red cell distribution width-standard deviation.

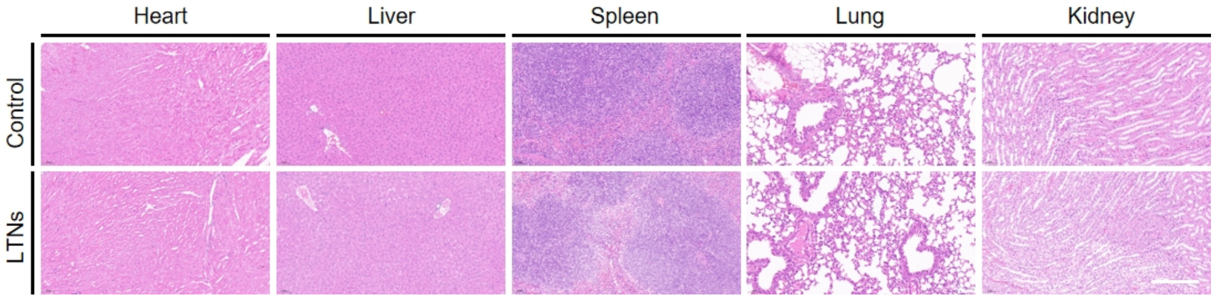

Figure S9. Hematoxylin and eosin-stained histological images of major organs from mice receiving LTNs at a mRNA dose of 0.5 mg kg^{-1}. Scale bar, 200 μm.

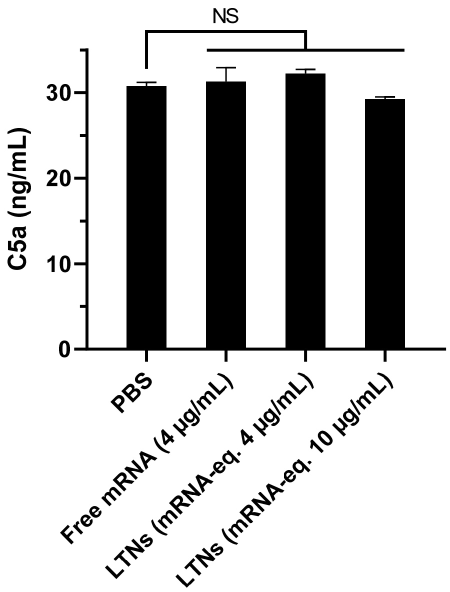

Figure S10. The level of human complement component 5a (C5a) in human serum treated with free mRNA or LTNs.

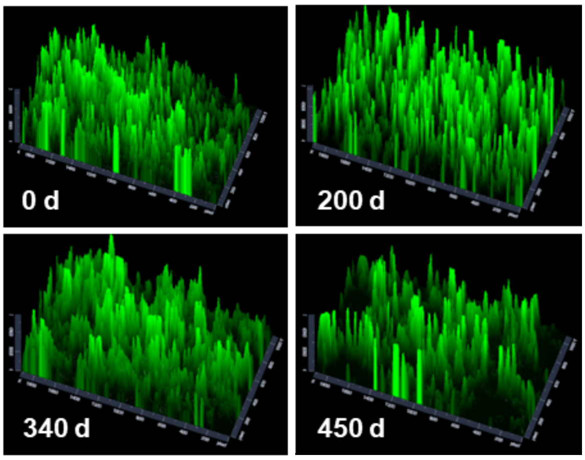

Figure S11. Assessment of the long-term stability of lung-targeted nanocarriers containing equimolar qtB-UC18 and DOPE after 200, 340, and 450 days of storage at ambient temperature. The delivery performance was evaluated by encapsulating eGFP mRNA into stored nanocarriers and monitoring the green fluorescent signals originating from 293T cells after 48 h of treatment.
